# Stereotactic Body Radiation Therapy for Oligometastatic Recurrent Esophageal Squamous Cell Carcinoma: A Retrospective Cohort Study From a Single Tertiary Center

**DOI:** 10.1002/cnr2.70248

**Published:** 2025-06-16

**Authors:** Atsuto Katano, Masanari Minamitani, Shingo Ohira, Subaru Sawayanagi, Hideomi Yamashita

**Affiliations:** ^1^ Department of Radiology The University of Tokyo Hospital Tokyo Japan; ^2^ Department of Radiological Science Faculty of Health Sciences, Tokyo Metropolitan University Tokyo Japan

**Keywords:** esophageal cancer, oligometastatic disease, stereotactic body radiation therapy

## Abstract

**Background:**

Oligometastatic disease, characterized by a limited number of metastatic lesions, has gained significant attention for its potential to enable long‐term survival with definitive local therapies. Esophageal cancer, an aggressive malignancy often diagnosed at advanced stages, carries a poor prognosis, particularly in cases involving distant metastasis. Stereotactic body radiation therapy (SBRT) has emerged as a promising local treatment modality for oligometastatic disease, offering precise high‐dose radiation delivery. This study evaluated the outcomes of SBRT in patients with oligometastatic esophageal cancer.

**Methods:**

This retrospective study analyzed 33 patients with esophageal cancer who underwent SBRT for all oligometastatic lesions at a single institution between August 2014 and March 2024. The inclusion criteria were patients with squamous cell carcinoma, extracranial oligometastases, and no concurrent systemic therapy. Oligometastatic diseases were categorized into de novo, repeat, and induced subtypes. Survival outcomes, including overall survival (OS) and progression‐free survival (PFS), were assessed using the Kaplan–Meier method.

**Results:**

The study cohort included 33 patients with a median age of 68 years. Most patients had one treated lesion, with SBRT doses ranging from 25–50 Gy in 4–10 fractions. Median OS and PFS were 17.3 months and 4.2 months, respectively. Patients with locoregional recurrence exhibited longer median PFS (5.1 months) and OS (19.6 months) compared to those with distant metastases (PFS: 3.0 months; OS: 14.8 months). Stratification by oligometastatic subtype revealed the best outcomes in de novo cases, with a median OS of 19.6 months and PFS of 8.8 months. Local control at 1 year was 90.7% for the entire cohort, with limited severe late adverse events.

**Conclusion:**

SBRT demonstrated promising outcomes in patients with oligometastatic esophageal cancer, offering high local control with minimal toxicity. Although these results highlight the feasibility of SBRT, larger prospective studies are needed to validate these findings.

## 1 Introduction

The concept of oligometastatic disease, characterized by a limited number of metastatic lesions, has garnered attention in cancer treatment [1, 2, 3]. This condition is believed to offer the potential for long‐term survival through definitive local therapies targeting metastatic lesions, as demonstrated historically in cases such as liver metastases from colorectal cancer [4]. Fong et al. reported that liver resection for colorectal metastases in the context of oligometastasis is both safe and effective and is the only potentially curative therapy for metastatic colorectal cancer [5]. Their study showed favorable clinical outcomes, with a 5‐year survival rate of 38%.

Esophageal cancer, owing to its aggressive nature and advanced stage at diagnosis, remains a malignancy with a poor prognosis [6]. Many patients are diagnosed at locally advanced or metastatic stages, making early detection challenging. Consequently, the median overall survival (OS) is low, with 5‐year survival rates below 20% [6]. For patients with distant metastases, treatment often remains palliative, and curative options are scarce. However, for esophageal cancer cases with identifiable oligometastatic disease, definitive local therapy may improve prognosis.

Stereotactic body radiation therapy (SBRT), a treatment modality that delivers high‐dose radiation with exceptional precision, is a promising option for the treatment of oligometastatic disease [7, 8, 9]. In particular, local treatments such as SBRT for oligometastases in the lungs or liver, particularly from esophageal cancer, have been reported to provide good clinical control [10, 11]. Nonetheless, given the poor prognosis associated with esophageal cancer, proper selection of patients with oligometastatic disease, standardization of treatment approaches, and accumulation of evidence through follow‐up studies are crucial.

This study aimed to report the treatment outcomes of patients with oligometastatic esophageal cancer treated at our institution, contributing to the growing evidence supporting the role of SBRT in this context.

## 2 Method

This was a retrospective, single‐institution study conducted at the Department of Radiology, The University of Tokyo Hospital. Due to the retrospective nature of the study, the requirement for informed consent was waived. The use of clinical data was approved by the Institutional Review Board of The University of Tokyo Hospital (IRB No. 3372–7). We retrospectively analyzed patients with esophageal cancer who underwent SBRT for all oligometastatic lesions as part of a post‐recurrence treatment between August 2010 and March 2024. This study was conducted in compliance with ethical guidelines, including the principles outlined in the Declaration of Helsinki. The inclusion criteria were: [1] recurrence following curative treatment (surgery or concurrent chemoradiotherapy), [2] pathologically confirmed squamous cell carcinoma at initial treatment, [3] SBRT performed on all oligometastatic lesions, [4] oligometastatic lesions located extracranially, [5] no concurrent systemic therapy (immunocheckpoint inhibitor, chemotherapy or molecular targeted therapy), and [6] availability of sufficient clinical records for evaluation. Only the first session was included in the analysis of patients who underwent multiple SBRT sessions.

SBRT was performed for patients with all oligometastatic lesions visible on imaging. The gross tumor volume (GTV) encompassed all radiologically detectable disease, and the clinical target volume (CTV) was defined as identical to the GTV. A planning target volume (PTV) was generated by expanding the GTV by 3–5 mm to account for setup uncertainties and organ motion. Dose and fractionation schedules were determined at the time of treatment based on tumor location, lesion size, and proximity to critical structures. The dose was prescribed to ensure that 95% of the PTV received the full prescription dose.

Oligometastatic disease was classified according to the consensus recommendations of the European Society for Radiotherapy and Oncology and the European Organization for Research and Treatment of Cancer [12]. In summary, oligometastatic disease was categorized into three subtypes: induced oligometastatic disease, defined as arising in patients with a history of polymetastatic disease; repeat oligometastatic disease, identified by a previous history of oligometastatic disease; and de novo oligometastatic disease, referring to a first‐time diagnosis. Following this definition, recurrences within 6 months of the initial diagnosis were classified as synchronous oligometastatic disease. Repeat oligometastases were defined as the recurrence of a limited number of metastatic lesions in patients who had previously achieved complete control of earlier oligometastatic disease through local therapies. Induced oligometastases referred to cases where patients initially presented with polymetastatic disease and subsequently developed a limited number of residual metastases after systemic therapy. Classification was determined based on each patient's prior disease history and treatment response.

Survival outcomes, including OS and progression‐free survival (PFS), were estimated using the Kaplan–Meier method, with time calculated from the first date of SBRT. OS was defined as the interval from therapy initiation to death from any cause. PFS was defined as the interval from the initiation of therapy to the first occurrence of radiological or clinical tumor progression or death from any cause.

Statistical analyses were conducted using the R statistical software package (R Foundation for Statistical Computing, Vienna, Austria). Owing to the limited sample size, survival curves were compared using the log‐rank test, because multivariate analysis was not feasible for generating statistically robust conclusions. Statistical significance was set at a P‐value of less than 0.05.

## 3 Result

Table 1 provides an overview of the 33 patients with oligometastatic disease. The median age was 68 years (range, 51–87 years), with 28 males and five females. The Karnofsky Performance Status was moderately favorable, ranging from 80 to 90, except in one patient. The initial anatomical segments included three cases in the cervical region, 27 in the thoracic region, and three in the abdominal region. The initial treatments consisted of surgery in 26 patients and definitive chemoradiotherapy in seven patients. The treated targets included 19 cases of locoregional recurrence, comprising one primary recurrence, five cervical lymph nodes, 11 thoracic lymph nodes, and two abdominal lymph nodes. Fourteen patients had distant metastases: five in the lungs, four in the liver, and five in other sites. Nineteen patients were diagnosed with de novo oligometastatic disease, including two with synchronous oligometastatic disease and 17 with metachronous oligometastatic recurrence. Eleven patients had repeat oligometastatic disease, including five cases of oligorecurrence and six of oligopersistence. Three patients were diagnosed with induced oligo‐persistence.

**TABLE 1:** Characteristics, treatment details, and recurrence patterns in patients with oligometastatic disease.

Variables	Number (%)
Age: median [range]	68 [51–87]
Gender	
Male	28 (85%)
Female	5 (15%)
Karnofsky performance status	
90	17 (52%)
80	15 (45%)
70	1 (3%)
Initial anatomical segments	
Cervical	3 (9%)
Thoracic	27 (82%)
Upper	5
Middle	15
Lower	7
Abdominal	3 (9%)
Initial treatment	
Surgery	26 (79%)
Chemoradiotherapy	7 (21%)
Recurrence pattern	
Locoregional	19 (58%)
Primary	1
Cervical lymph nodes	5
Thoracic lymph nodes	11
Abdominal lymph nodes	2
Distant metastasis	14 (42%)
Lung	5
Liver	4
Others	5
Oligometastatic disease	
De novo	19 (58%)
Synchronous oligometastatic	2
Metachronous oligorecurrence	17
Repeat	11 (33%)
Repeat oligorecurrence	5
Repeat oligopersistence	6
Induced	3 (9%)
Induced oligopersistence	3
Number of treated oligometastic lesion	
1	26 (79%)
2	6 (18%)
3	1 (3%)
Dose and fractionation	
50Gy in 10 fractions	19 (58%)
55 Gy in 4 fractions	4 (12%)
48 Gy in 4 fractions	3 (9%)
70 Gy in 10 fractions	2 (6%)
Others	5 (15%)
Interval from Initial diagnosis to SBRT	14.3 Months [2.3–140.8]

Most patients (79%) had one treated lesion. The dose and fractionation of SBRT were primarily 50 Gy in 10 fractions for most patients, followed by 55 Gy in four fractions, 48 Gy in four fractions, and 70 Gy in 10 fractions, with a few patients receiving other regimens. The median dose and fractionation were 50 Gy (range, 25–50 Gy) and 10 fractions (range, 4–10 fractions), respectively. The median biologically effective dose, calculated using the linear‐quadratic model with an alpha/beta ratio of 10 Gy, was 75 Gy (range 37.5–130.6 Gy). The median interval from initial diagnosis to SBRT was 14.3 months (range from 2.3 to 140.8 months).

**FIGURE 1:**
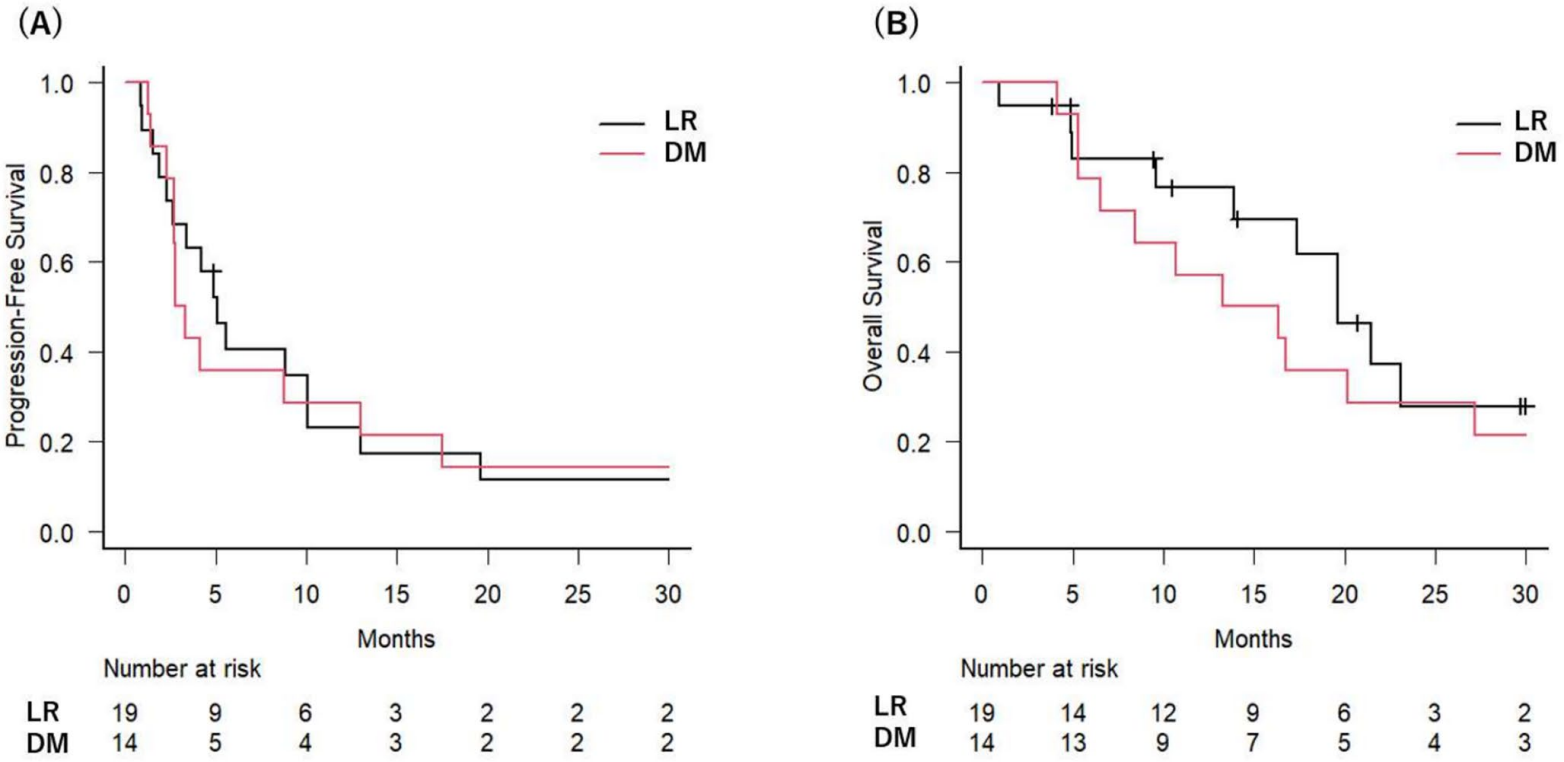
Survival outcomes after SBRT in patients with oligometastatic disease. Progression‐free survival (A) and overall survival (B), stratified by locoregional recurrence (LR) versus distant metastases (DM).

The median follow‐up period was 14.1 months (range: 1.0 to 92.1 months). The median OS and PFS for all 33 patients were 17.3 months (95% CI: 10.7–23.1 months) and 4.2 months (95% CI: 2.7–8.8 months), respectively. The 2‐year OS and PFS rates were 29.4% (95% CI: 13.7%–46.9%) and 12.9% (95% CI: 4.1%–27.0%), respectively. Among the 20 patients with locoregional recurrence, PFS tended to be better compared to that in the 15 patients with distant metastases, with 5.1 months (95% CI: 2.3–10.1 months) versus 3.0 months (95% CI: 2.3–13.0 months), and the difference was not statistically significant (*p* = 0.924) [Figure 1A]. A similar trend was observed for OS, with 19.6 months (95% CI: 9.6–N.A. months) for locoregional recurrence compared to 14.8 months (95% CI: 5.3–27.2 months) for distant metastases, and the difference was not statistically significant (*p* = 0.262) [Figure 1B].

**FIGURE 2:**
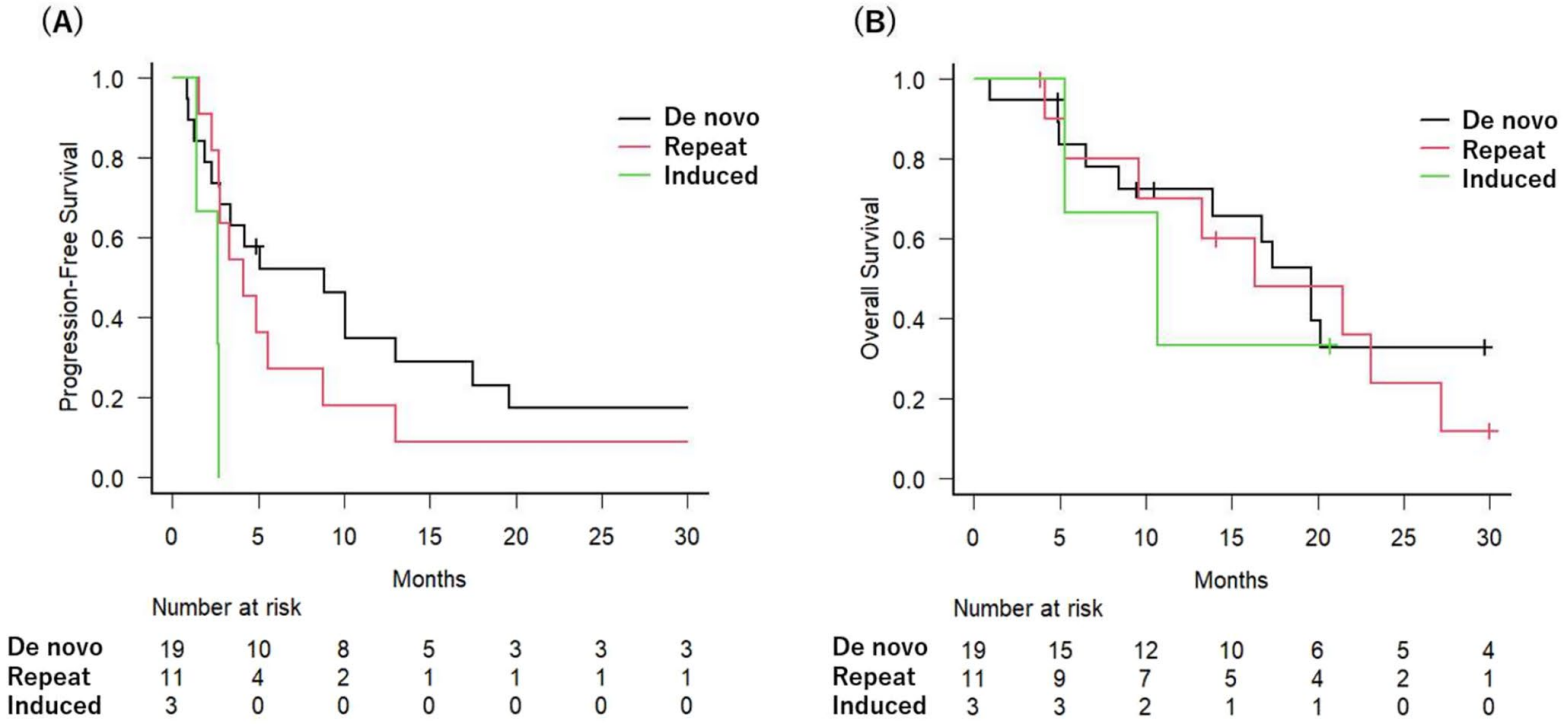
Survival outcomes based on SBRT Timing in patients with oligometastatic disease. Progression‐free survival (A) and overall survival (B) stratified according to de novo, repeat, and induced cases.

Stratified by oligometastatic classification, PFS was observed as follows: 8.8 months (95% CI: 2.3–17.5 months) for de novo cases, 4.1 months (95% CI: 2.3–8.7 months) for repeat cases, and 2.6 months (95% CI: 1.4–N.A. months) for induced cases [Figure 2A]. OS was 19.6 months (95% CI: 8.4–44.3 months), 16.3 months (95% CI: 4.1–27.2 months), and 10.7 months (95% CI: 5.3–N.A. months) for de novo, repeat, and induced cases, respectively [Figure 2B]. Given the limited sample size, multivariate Cox proportional hazards regression was not feasible in this study. However, exploratory univariate Cox regression analyses were performed to assess the impact of selected clinical factors, including age, location of metastasis, radiation dose, and oligometastatic subtype, on overall survival and progression‐free survival. The results of these analyses are presented in the supplementary table.

Of the 33 patients, 29 experienced disease progression during the observation period. The most frequent site of recurrence was the regional lymph nodes (11 patients), followed by the lungs (seven patients) and the liver (five patients). In most cases, disease progression occurred outside the SBRT treatment site. Local relapse after SBRT was found in four patients, resulting in a 1‐year local control rate of 90.7% (95% CI: 73.9%–96.9%). Regarding treatment‐related toxicity, Grade 2 pneumonitis was observed in three patients, and Grade 2 esophagitis was observed in one patient. No severe late adverse events were noted, except for one case of Grade 3 pneumonitis.

Salvage therapies after SBRT for oligometastatic disease included cytotoxic chemotherapy in 12 patients, best supportive care in nine, repeated radical local therapy in seven (concurrent chemoradiotherapy in two, surgery in one, and re‐SBRT in four), and immune checkpoint inhibitors in one.

## 4 Discussion

This study evaluated the clinical outcomes of SBRT for managing the oligometastatic locoregional recurrence of esophageal squamous cell carcinoma (ESCC) at a single tertiary center.

In cases of locoregional recurrence after curative treatment for esophageal cancer, salvage surgery or chemoradiotherapy (CRT) is generally considered the standard definitive treatment [13]. A meta‐analysis by Faiz et al. reported that salvage esophagectomy achieved a 3‐year OS rate of approximately 39% in patients with isolated local recurrence [14]. Furthermore, Mori et al. demonstrated that salvage treatments with curative intent, including esophagectomy, endoscopic therapies, and lymphadenectomy, yielded 5‐year survival rates of 47.4%, 70.9%, and 33.3%, respectively [15].

In the context of salvage CRT for recurrence after surgery, Yamashita et al. conducted a multi‐institutional retrospective study evaluating salvage radiotherapy for lymph node oligo‐recurrence in esophageal cancer, showing a 3‐year overall survival of 37% [16]. A recent retrospective study demonstrated that salvage concurrent chemoradiotherapy (CCRT) achieved a median overall survival of 30.1 months and a 2‐year OS rate of 57.4% in patients with loco‐regional recurrence of esophageal squamous cell carcinoma [17]. Jingu et al. evaluated recurrence patterns after salvage CRT for postoperative loco‐regional recurrent esophageal cancer, finding a 5‐year overall survival rate of 43.7% and that most failures occurred within 42 months [18].

Compared with these outcomes, the present study demonstrated a 2‐year OS rate of approximately 30% in patients with locoregional recurrence treated with SBRT. This suggests that SBRT alone may yield inferior survival outcomes compared to standard salvage surgery or CRT in selected populations. However, it is important to note that many patients in our cohort were likely ineligible for surgery or intensive CRT due to comorbidities, impaired performance status, or prior treatment‐related toxicity. For such patients, SBRT served as a minimally invasive alternative with acceptable local control and a favorable safety profile.

Our findings suggest that SBRT is a promising therapeutic option with outcomes comparable to those reported in previous studies. Seyedin et al. demonstrated the feasibility of SBRT as salvage therapy for locoregional recurrence of esophageal cancer after curative‐intent treatment, reporting a median PFS of 5.0 months and median OS of 12.9 months [19].

Metastasis‐directed therapy for oligometastasis has gained increasing attention. A cornerstone in the management of oligometastases is the SABR‐COMET trial, which demonstrated that stereotactic ablative radiotherapy (SABR) significantly improves OS and PFS in patients with oligometastatic disease [20]. The 8‐year OS was significantly higher in the SABR arm at 27.2% compared to 13.6% in the control arm (hazard ratio, 0.50; 95% CI, 0.30–0.84; *p* = 0.008). Similarly, 8‐year PFS estimates were 21.3% in the SABR arm versus 0.0% in the control arm (hazard ratio, 0.45; 95% CI, 0.28–0.72; *p* < 0.001). Oligometastatic disease management has been actively explored and implemented for cancers such as non‐small cell lung cancer and prostate cancers [21, 22].

Several studies have reported the utility of local therapy for the treatment of oligometastatic esophageal cancer. A phase 2 trial assessing SBRT followed by a median of four cycles of chemotherapy for patients with oligometastatic ESCC reported a median PFS of 13.3 months [23]. The ESO‐Shanghai 13 trial, an open‐label randomized phase 2 trial, showed that combining local and systemic therapies significantly improves PFS [24]. Median PFS was 15.3 months in the systemic and local therapy groups compared to 6.4 months in the systemic therapy‐only group, with a hazard ratio of 0.26.

These trials emphasized the benefits of combining local and systemic therapies. In contrast, our study specifically focused on the outcomes of SBRT administered without concurrent systemic therapy. The major advantage of using SBRT alone is the avoidance of systemic toxicities, which helps preserve patient quality of life while maintaining control over limited disease burden. However, omitting systemic therapy may increase the risk of occult micrometastatic progression, raising an important clinical question about when local therapy alone is sufficient. Although excellent local control was achieved, the emergence of new oligometastatic or polymetastatic lesions remained a significant challenge. According to the European Clinical Practice Guidelines for Oligometastatic Esophagogastric Cancer, upfront local treatment combined with systemic therapy is recommended for patients with metachronous oligometastases and a disease‐free interval exceeding 2 years [25].

However, little is known about the molecular mechanisms underlying oligometastatic disease and the pathways that differentiate polymetastatic from oligometastatic spread [26]. Recent studies have identified associations between oligometastatic behavior and various molecular and cellular factors, including distinct microRNA profiles, chromosomal alterations, and driver gene mutations [27]. Advancing our understanding of the molecular and biological features of oligometastatic diseases is essential to improve the distinction between metastatic behaviors and enable the development of precision treatments.

## 5 This Study Had Several Limitations

First, as this was a retrospective analysis conducted at a single institution, there was an inherent risk of selection bias. Second, the sample size was relatively small, which limited the statistical power and generalizability of the findings. Third, the lack of a control group precludes definitive conclusions regarding the comparative efficacy of SBRT. Furthermore, the heterogeneity of the cohort, including differences in initial treatments, metastatic sites, and SBRT dose regimens, may have introduced additional confounding factors and made direct comparisons challenging. This heterogeneity must be acknowledged as a limitation that could have influenced the interpretation of the results. Nevertheless, it also reflects the diversity encountered in real‐world clinical practice, and we believe that including a wide range of clinical scenarios may enhance the applicability of our findings to routine patient care.

## 6 Conclusion

SBRT is a feasible and effective treatment option for patients with oligometastatic locoregional recurrence of ESCC, offering good local control and an acceptable toxicity profile. While these results are promising, larger studies with longer follow‐up are essential to validate these findings and establish SBRT as a standard treatment modality in this setting.

## Author Contributions

Conceptualization: Atsuto Katano, Masanari Minamitani. Methodology: Atsuto Katano, Masanari Minamitani, Shingo Ohira. Software: Shingo Ohira. Validation: Atsuto Katano, Shingo Ohira, Subaru Sawayanagi. Formal analysis: Atsuto Katano, Masanari Minamitani. Investigation: Atsuto Katano, Subaru Sawayanagi, Masanari Minamitani. Resources: Hideomi Yamashita, Subaru Sawayanagi. Data curation: Subaru Sawayanagi, Masanari Minamitani. Visualization: Atsuto Katano, Shingo Ohira. Writing – original draft preparation: Atsuto Katano. Writing – review and editing: Masanari Minamitani, Shingo Ohira, Subaru Sawayanagi, Hideomi Yamashita. Supervision: Hideomi Yamashita, Atsuto Katano. Project administration: Atsuto Katano. Final approval of manuscript: All authors.

## Ethics Statement

This study was conducted in compliance with ethical guidelines and approved by the Institutional Review Board of the Research Ethics Committee, Faculty of Medicine, University of Tokyo (approval number: 3372–7).

## Conflicts of Interest

The authors declare no conflicts of interest.

## Supporting information


**Supplementary Table 1** Univariate Cox proportional hazard analysis of PFS and OS

## Data Availability

The data that support the findings of this study are available on request from the corresponding author. The data are not publicly available due to privacy or ethical restrictions.

## References

[1] W. Liu , H. Bahig , and D. A. Palma , “Oligometastases: Emerging Evidence,” Journal of Clinical Oncology 40, no. 36 (2022): 4250–4260, 10.1200/jco.22.01482. 36306497

[2] P. Pacifico , R. R. Colciago , F. De Felice , et al., “A Critical Review on Oligometastatic Disease: A Radiation Oncologist's Perspective,” Medical Oncology 39, no. 12 (2022): 181, 10.1007/s12032-022-01788-8. 36071292 PMC9452425

[3] S. Hellman and R. R. Weichselbaum , “Oligometastases,” Journal of Clinical Oncology 13, no. 1 (1995): 8–10, 10.1200/jco.1995.13.1.8. 7799047

[4] H. D. González and J. Figueras , “Practical Questions in Liver Metastases of Colorectal Cancer: General Principles of Treatment,” HPB: The Official Journal of the International Hepato Pancreato Biliary Association 9, no. 4 (2007): 251–258, 10.1080/13651820701457992. 18345300 PMC2215392

[5] Y. Fong , A. M. Cohen , J. G. Fortner , et al., “Liver Resection for Colorectal Metastases,” Journal of Clinical Oncology 15, no. 3 (1997): 938–946, 10.1200/jco.1997.15.3.938. 9060531

[6] M. Sheikh , G. Roshandel , V. McCormack , and R. Malekzadeh , “Current Status and Future Prospects for Esophageal Cancer,” Cancers (Basel) 15, no. 3 (2023): 765, 10.3390/cancers15030765. 36765722 PMC9913274

[7] A. Katano , M. Minamitani , and S. Ohira , “Yamashita H. Recent Advances and Challenges in Stereotactic Body Radiotherapy,” Technology in Cancer Research & Treatment 23 (2024): 15330338241229363, 10.1177/15330338241229363. 38321892 PMC10851756

[8] F. Alongi , S. Arcangeli , A. R. Filippi , U. Ricardi , and M. Scorsetti , “Review and Uses of Stereotactic Body Radiation Therapy for Oligometastases,” Oncologist 17, no. 8 (2012): 1100–1107, 10.1634/theoncologist.2012-0092. 22723509 PMC3425528

[9] B. E. Onderdonk , S. I. Gutiontov , and S. J. Chmura , “The Evolution (And Future) of Stereotactic Body Radiotherapy in the Treatment of Oligometastatic Disease,” Hematology/Oncology Clinics of North America 34, no. 1 (2020): 307–320, 10.1016/j.hoc.2019.09.003. 31739951

[10] T. Yamamoto , Y. Niibe , Y. Matsumoto , et al., “Stereotactic Body Radiotherapy for Pulmonary Oligometastases From Esophageal Cancer: Results and Prognostic Factors,” Anticancer Research 40, no. 4 (2020): 2065–2072, 10.21873/anticanres.14164. 32234898

[11] A. Katano , H. Yamashita , and K. Nakagawa , “Stereotactic Body Radiotherapy for Oligo‐Recurrence in the Liver in a Patient With Esophageal Carcinoma: A Case Report,” Molecular and Clinical Oncology 7, no. 6 (2017): 1061–1063, 10.3892/mco.2017.1441. 29285374 PMC5740860

[12] M. Guckenberger , Y. Lievens , A. B. Bouma , et al., “Characterisation and Classification of Oligometastatic Disease: A European Society for Radiotherapy and Oncology and European Organisation for Research and Treatment of Cancer Consensus Recommendation,” Lancet Oncology 21, no. 1 (2020): e18–e28, 10.1016/S1470-2045(19)30718-1. 31908301

[13] M. T. Corkum , M. K. Buyyounouski , A. J. Chang , et al., “Salvage Prostate Brachytherapy in Radiorecurrent Prostate Cancer: An International Delphi Consensus Study,” Radiotherapy and Oncology 184 (2023): 109672, 10.1016/j.radonc.2023.109672. 37059334

[14] Z. Faiz , W. P. M. Dijksterhuis , J. G. M. Burgerhof , et al., “A Meta‐Analysis on Salvage Surgery as a Potentially Curative Procedure in Patients With Isolated Local Recurrent or Persistent Esophageal Cancer After Chemoradiotherapy,” European Journal of Surgical Oncology 45, no. 6 (2019): 931–940, 10.1016/j.ejso.2018.11.002. 30447937

[15] K. Mori , K. Sugawara , S. Aikou , et al., “Esophageal Cancer Patients' Survival After Complete Response to Definitive Chemoradiotherapy: A Retrospective Analysis,” Esophagus 18, no. 3 (2021): 629–637, 10.1007/s10388-021-00817-1. 33625649

[16] H. Yamashita , K. Jingu , Y. Niibe , et al., “Definitive Salvage Radiation Therapy and Chemoradiation Therapy for Lymph Node Oligo‐Recurrence of Esophageal Cancer: A Japanese Multi‐Institutional Study of 237 Patients,” Radiation Oncology 12, no. 1 (2017): 38, 10.1186/s13014-017-0780-5. 28219406 PMC5319190

[17] A. Katano , T. Kiritoshi , S. Sawayanagi , and H. Yamashita , “Salvage Chemoradiotherapy for Loco‐Regional Recurrence of Esophageal Squamous Cell Carcinoma After Esophagectomy,” Journal of Clinical Medicine 14, no. 5 (2025): 1540, 10.3390/jcm14051540. 40095468 PMC11899801

[18] K. Jingu , R. Umezawa , T. Yamamoto , et al., “Patterns of Failure After Salvage Chemoradiotherapy for Postoperative Loco‐Regional Recurrent Esophageal Cancer: 20‐Year Experience in a Single Institution in Japan,” Esophagus 19, no. 4 (2022): 639–644, 10.1007/s10388-022-00922-9. 35575821

[19] S. N. Seyedin , M. K. Gannon , K. A. Plichta , et al., “Safety and Efficacy of Stereotactic Body Radiation Therapy for Locoregional Recurrences After Prior Chemoradiation for Advanced Esophageal Carcinoma,” Frontiers in Oncology 10 (2020): 1311, 10.3389/fonc.2020.01311. 32850412 PMC7412633

[20] M. Van Oirschot , A. Bergman , W. Verbakel , et al., “Determining Planning Priorities for SABR for Oligometastatic Disease: A Secondary Analysis of the SABR‐COMET Phase II Randomized Trial,” International Journal of Radiation Oncology, Biology, Physics 114, no. 5 (2022): 1016–1021, 10.1016/j.ijrobp.2022.01.002. 35031340

[21] L. Nicholls , E. Chapman , V. Khoo , et al., “Metastasis‐Directed Therapy in Prostate Cancer: Prognostic Significance of the ESTRO/EORTC Classification in Oligometastatic Bone Disease,” Clinical Oncology (Royal College of Radiologists) 34, no. 1 (2022): 63–69, 10.1016/j.clon.2021.10.004. 34756755

[22] M. V. Infante and T. Berghmans , “Oligometastatic Non‐Small Cell Lung Cancer: From Biology to Clinical Practice,” Translational Lung Cancer Research 10 (2021): 3320–3323. 34430368 10.21037/tlcr-21-533 PMC8350091

[23] Q. Liu , Z. Zhu , Y. Chen , et al., “Phase 2 Study of Stereotactic Body Radiation Therapy for Patients With Oligometastatic Esophageal Squamous Cell Carcinoma,” International Journal of Radiation Oncology, Biology, Physics 108, no. 3 (2020): 707–715, 10.1016/j.ijrobp.2020.05.003. 32417405

[24] Q. Liu , J. Chen , Y. Lin , et al., “Systemic Therapy With or Without Local Intervention for Oligometastatic Oesophageal Squamous Cell Carcinoma (ESO‐Shanghai 13): An Open‐Label, Randomised, Phase 2 Trial,” Lancet Gastroenterology & Hepatology 9, no. 1 (2024): 45–55, 10.1016/s2468-1253(23)00316-3. 37980921

[25] T. E. Kroese , S. Bronzwaer , P. S. N. van Rossum , et al., “European Clinical Practice Guidelines for the Definition, Diagnosis, and Treatment of Oligometastatic Esophagogastric Cancer (OMEC‐4),” European Journal of Cancer 204 (2024): 114062, 10.1016/j.ejca.2024.114062. 38678762

[26] A. Uppal , M. K. Ferguson , M. C. Posner , S. Hellman , N. N. Khodarev , and R. R. Weichselbaum , “Towards a Molecular Basis of Oligometastatic Disease: Potential Role of Micro‐RNAs,” Clinical & Experimental Metastasis 31, no. 6 (2014): 735–748, 10.1007/s10585-014-9664-3. 24968866 PMC4138440

[27] A. Ottaiano , M. Santorsola , L. Circelli , et al., “Oligo‐Metastatic Cancers: Putative Biomarkers, Emerging Challenges and New Perspectives,” Cancers (Basel) 15, no. 6 (2023): 1827, 10.3390/cancers15061827. 36980713 PMC10047282

