# Supplementary material for: Stereotactic Body Radiation Therapy for Oligometastatic Recurrent Esophageal Squamous Cell Carcinoma: A Retrospective Cohort Study From a Single Tertiary Center
Source: Cancer Rep (Hoboken). 2025 Jun 16;8(6):e70248. doi: 10.1002/cnr2.70248 (PMC12170229; doi:10.1002/cnr2.70248)
Supplement: Supplementary file 1 — Supplementary Table 1 Univariate Cox proportional hazard analysis of PFS and OS [file CNR2-8-e70248-s001.docx]

Supplementary Table 1: Univariate Cox proportional hazard analysis of PFS and OS

|  |  | PFS | | |  | OS | | |
| --- | --- | --- | --- | --- | --- | --- | --- | --- |
| Covariables |  | Hazard ratio | 95% CI | p value |  | Hazard ratio | 95% CI | p value |
| Age | ≤ 70 vs > 70 years old | 1.306 | 0.640 - 2.665 | 0.462 |  | 0.833 | 0.358 - 1.940 | 0.672 |
| Gender | Male vs Female | 1.828 | 0.623 - 5.365 | 0.272 |  | 4.636 | 0.918 - 23.410 | 0.063 |
| Performance status | ≥ 90 vs < 90 | 1.951 | 0.914 - 4.165 | 0.084 |  | 1.680 | 0.731 - 3.859 | 0.222 |
| Location of metastasis | Locoregional vs distant | 0.965 | 0.469 - 1.986 | 0.924 |  | 1.600 | 0.698 - 3.666 | 0.267 |
| Prescription dose (Gy) | ≥ 100 vs < 100 | 0.685 | 0.320 - 1.468 | 0.331 |  | 1.135 | 0.493 - 2.611 | 0.767 |
| Oligometastatic type | De novo vs others | 1.947 | 0.921 - 4.116 | 0.081 |  | 1.205 | 0.524 - 2.769 | 0.661 |

Prescription dose (≥100 Gy vs <100 Gy, based on the biologically effective dose calculated assuming an α/β ratio of 10)
